# Generation and evaluation of an indicator of the health system’s performance in maternal and reproductive health in Colombia: An ecological study

**DOI:** 10.1371/journal.pone.0180857

**Published:** 2017-08-30

**Authors:** Carlos Eduardo Pinzón-Flórez, Julian Alfredo Fernandez-Niño, Luz Mery Cardenas-Cardenas, Diana Marcela Díaz-Quijano, Myriam Ruiz-Rodriguez, Ludovic Reveiz, Armando Arredondo-López

**Affiliations:** 1 Grupo de Investigación en Salud, Universidad de La Sabana, Chía, Colombia; 2 Departamento de Salud Pública, Universidad del Norte, Barranquilla, Colombia; 3 Grupo de investigación municipio saludable. Universidad Pedagógica y Tecnológica de Colombia. Tunja, Colombia; 4 Observatorio Nacional de Salud. Instituto Nacional de Salud, Bogotá, Colombia; 5 Departamento de Salud Pública, Universidad Industrial de Santander. Bucaramanga, Colombia; 6 Pan American Health Organization, Washington, D.C., United States of America; 7 Centro de investigación de sistemas de salud. Instituto Nacional de Salud Pública, Cuernavaca (Morelos), México; Georgia Institute of Technology, UNITED STATES

## Abstract

**Objective:**

To generate and evaluate an indicator of the health system’s performance in the area of maternal and reproductive health in Colombia.

**Materials and methods:**

An indicator was constructed based on variables related to the coverage and utilization of healthcare services for pregnant and reproductive-age women. A factor analysis was performed using a polychoric correlation matrix and the states were classified according to the indicator’s score. A path analysis was used to evaluate the relationship between the indicator and social determinants, with the maternal mortality ratio as the response variable.

**Results:**

The factor analysis indicates that only one principal factor exists, namely "coverage and utilization of maternal healthcare services" (eigenvalue 4.35). The indicator performed best in the states of Atlantic, Bogota, Boyaca, Cundinamarca, Huila, Risaralda and Santander (Q4). The poorest performance (Q1) occurred in Caqueta, Choco, La Guajira, Vichada, Guainia, Amazonas and Vaupes. The indicator’s behavior was found to have an association with the unsatisfied basic needs index and women’s education (*β* = -0.021; 95%CI -0031 to -0.01 and *β* 0.554; 95%CI 0.39 to 0.72, respectively). According to the path analysis, an inverse relationship exists between the proposed indicator and the behavior of the maternal mortality ratio (*β* = -49.34; 95%CI -77.7 to -20.9); performance was a mediating variable.

**Discussion:**

The performance of the health system with respect to its management of access and coverage for maternal and reproductive health appears to function as a mediating variable between social determinants and maternal mortality in Colombia.

## Introduction

At the beginning of this century, Colombia was among 189 countries to adopt the Millennium Development Declaration, which included a commitment to fulfill eight objectives. One of those objectives is to improve maternal health (Millennium Development Objective (MDO) # 5) [1, 2]. Maternal mortality was chosen as the primary health outcome to evaluate progress towards achieving this objective, with the goal of a reduction of 75% in the maternal mortality ratio (MMR) from 1990 to 2015. To this end, renewing the enrollment of vulnerable populations in health services was proposed as well as substantially improving access to health care [1].

In 2005, the global maternal mortality ratio was 400 per 100,000 live births [3,4], having decreased 0,37% annually since the year 2000. By 2013, this had decreased even further to 209.1 per 100,000 live births, representing a 1,3% annual decrease. Meanwhile, in a separate analysis Hill and colleagues [5] estimated a 2,5% annual reduction in the maternal mortality ratio for 125 countries over the 5-year period from 2000 to 2005 [5]. Nevertheless, notable differences exist in the maternal mortality ratio between high-income countries versus low- and medium-income countries, which reflect health inequities [6]. In 1990, the ratio was 24.5 per 100,000 live births in developed countries versus 317.6 per 100,000 live births in developing countries. These disparities continued through 2013, when the ratio was 12.1 per 100,000 live births and 232.8 per 100,000 live births, respectively [7, 8].

These differences continue to be observed regardless of whether maternal mortality is measured based on absolute numbers or risk of maternal death. The lowest estimate of risk of maternal death is roughly 1 in 30,000 (Sweden) while the highest is 1 in 6 (Afghanistan and Sierra Leone) [9,10].

In Colombia, although the mortality ratio has decreased over the past 10 years, 88,2 per 100,000 live births in 1998 to and 69,3 per 100,000 live births by 2011, it continues to be higher than reports from developed countries [7, 11], The second national health report by the National Health Observatory analyzed possible causes of maternal mortality and its trends in Colombia for the years of 1998 to 2011, the states of Choco, Cauca, La Guajira, Magdalena, Nariño, and Caqueta was higher than the national average [12]. In 2011, the highest number of absolute deaths occurred in Bogota, although it has one of the lowest MMR [13].

On the other hand, evidence from the scientific literature shows that providing quality health services can prevent maternal death due to complications [14–17]. This requires establishing primary health care programs such as family planning, and psychosocially preparing the mother and father for pregnancy. Quality health services also involve measures to ensure that pregnancy progresses adequately, such as timely access to healthcare services provided by qualified healthcare professionals and the availability of resources needed for treatment. In addition, the "three delays” model developed by Columbia University is very helpful to identify direct and indirect causes of maternal death as well as possible barriers to accessing and utilizing maternal health services [18].

Maternal mortality is sensitive to social inequity and socioeconomic marginalization. Its incidence has been observed to be higher in regions with less economic capital (lower per capita income, rural regions, educational level) [19, 20]. Also individual factors associated with the risk of death during pregnancy also have been identified, such as low socioeconomic status, lack of access to timely and quality health services, and cultural differences of minority populations that contribute to rejecting healthcare treatment [19].

In 2004, the Colombian Ministry of Health and Social Protection recognized the seriousness of the situation and established an action plan to address maternal mortality. Nevertheless, the measures proposed have not been sufficient. Coverage by regional insurance agencies has been limited and the plan has had little impact [21].

The healthcare system’s coverage of primary healthcare services for pregnancy and delivery needs to be evaluated in order to make informed, evidence-based decisions [22]. The importance of the assessment of maternal health as an indicator of the performance of healthcare systems has been demonstrated by the decrease in maternal mortality resulting from interventions implemented by the system, such as the institutionalization of delivery, professionalization of delivery care and the implementation of primary care programs [23]. Therefore, it is useful to construct an indicator of the performance of maternal and reproductive health at the state level in Colombia, which could be implemented to identify inequalities within and among countries (regions) and thereby prioritize public policies and identify the healthcare system reforms that are needed.

## Materials and methods

### Type of study and sources of information

An ecological, cross-sectional study of several groups was conducted in Colombia at the state level. The study was performed based on information from the National Administrative Department of Statistics (DANE, Spanish acronym) [24,25], the National Planning Department (DNP, Spanish acronym), the Ministry of Health and Social Protection, and with data from the 2010 National Demographics Health Survey (ENDS, Spanish acronym) [26, 27]. The latter is representative of Colombia both nationally and at the state level, and included 101,482 women of reproductive age in 256 municipalities in the 33 states [27]. The ENDS collected information related to coverage and utilization of maternal health services in the areas of family planning, prenatal check-ups, delivery care and education of the expectant mother. Data concerning social determinants (unsatisfied basic needs) at the state level for 2010 were obtained from the DNP [28], the DANE, [25] and the Ministry of Health and Social Protection. S1 Appendix.

### Variables

#### Response variable

The response variable was the MMR for the year 2011. Maternal death was defined according to the International Classification of Diseases version 10 (ICD-10) [29]. All maternal deaths in 2011 classified with the following diagnoses were included: pregnancy with abortive outcome (O00-O08), edema, proteinuria and hypertensive disorders in pregnancy, childbirth and the puerperium (O10-O16), other maternal disorders predominantly related to pregnancy (O20-O29), maternal care related to the fetus and amniotic cavity and possible delivery problems (O30-O48), complications of labor and delivery (O60-O75), complications predominantly related to the puerperium (O85-O92), other obstetric conditions not classified elsewhere (O95-O99), and causes specified in other chapters (A34, B20-B24, C58, D392, E230, F530-F539, M830). Excluded were deaths that occurred in another country and cases in which information about the municipality or time of death was missing.

#### Variables related to coverage and utilization of services

The variables *coverage* and *utilization of services* for each state, for the year 2010, included: percentage of deliveries in healthcare institutions, percentage of reproductive-age women who at the time of the ENDS survey used some type of modern birth control, percentage of women who delivered within 5 years of the survey and did not receive prenatal care, percentage of deliveries by a healthcare professional, percentage of pregnant women for whom the cost of prenatal care was not covered due to not being enrolled, percentage of pregnant women for whom the cost of the delivery was not covered due to not being enrolled, percentage of pregnant women for whom the cost of postnatal care was not covered due to not being enrolled, percentage of deliveries by a midwife or other person or family member [27].

#### Social determinant variables

Indicators of social determinants were selected based on two criteria: 1) relevance and 2) feasibility. Relevance refers to scientific evidence that supports an association between social determinants of health and maternal health. Feasibility refers to the availability of state- and municipal-level information related to the indicators. Seven indicators were identified: Gini Coefficient, Human Development Index, unsatisfied basic needs, education of the mother, multidimensional poverty index, corruption index and effective access to health services. The corruption index was excluded as an indicator because it did not for meet the relevance criterion; the Gini coefficient, human development index, index of multidimensional poverty index, corruption index and the effective access to health services indicator did not meet the feasibility criterion due to a lack of data for all observation periods.

The social variables included the education of the mother and the unsatisfied basic needs index. These two indicators have been evaluated in other regional and economic contexts in terms of their association with maternal health, child health outcomes and the performance of health systems in the areas of delivery and the quality of health services [30–32]. These indicators are generated periodically at the state level. Data from the year 2010 were used for the present study. The education of the mother was defined as the percentage of mothers who finished secondary school, standardized by municipality and state. The unsatisfied basic needs index is a compound indicator composed of indicators related to housing conditions, such as: housing materials, access to public services, extreme overcrowding, economic dependence and access to education. According to the unsatisfied basic needs index, a poor home is one whose basic needs are not met in terms of at least one indicator [25]. This index is constructed using a factor analysis and has been widely used in Colombia.

### Statistical analysis

The units of analysis were the 33 states. The indicator of coverage and utilization of maternal health services was constructed using a polychoric correlation index with the maternal healthcare services variables mentioned earlier. A factor analysis was conducted using the principal component factor extraction method. The resulting indicator was then categorized by quartiles and the states were scored based on these categories. Finally, a path analysis (a type of structural equation model (SEM)) [30–32] was applied in order to evaluate the hypothesis that a relationship exists among social determinants, the indicator of the quality of healthcare services and health outcomes. The present study used a recursive path analysis given the characteristics of the proposed model, in which the health system is a mediating factor between the social determinants of health and health outcomes [33–35]. The main characteristics of the recursive path analysis are that there are no cycles or reciprocal associations among the variables and no correlation with errors related to the dependent variables. The theoretical model for the path analysis is given by:

Y=BY+ΓX+ε

Where:

Y = vector of p x 1 dependent variables observed
X = vector of q x 1 independent variables observed
B = matrix of p x p coefficients corresponding to Y
Γ = matrix of p x q corresponding to X
ε = vector of p x 1 errors.

#### Model of relationships among social determinants, health system performance and health outcomes

**Fig 1:**
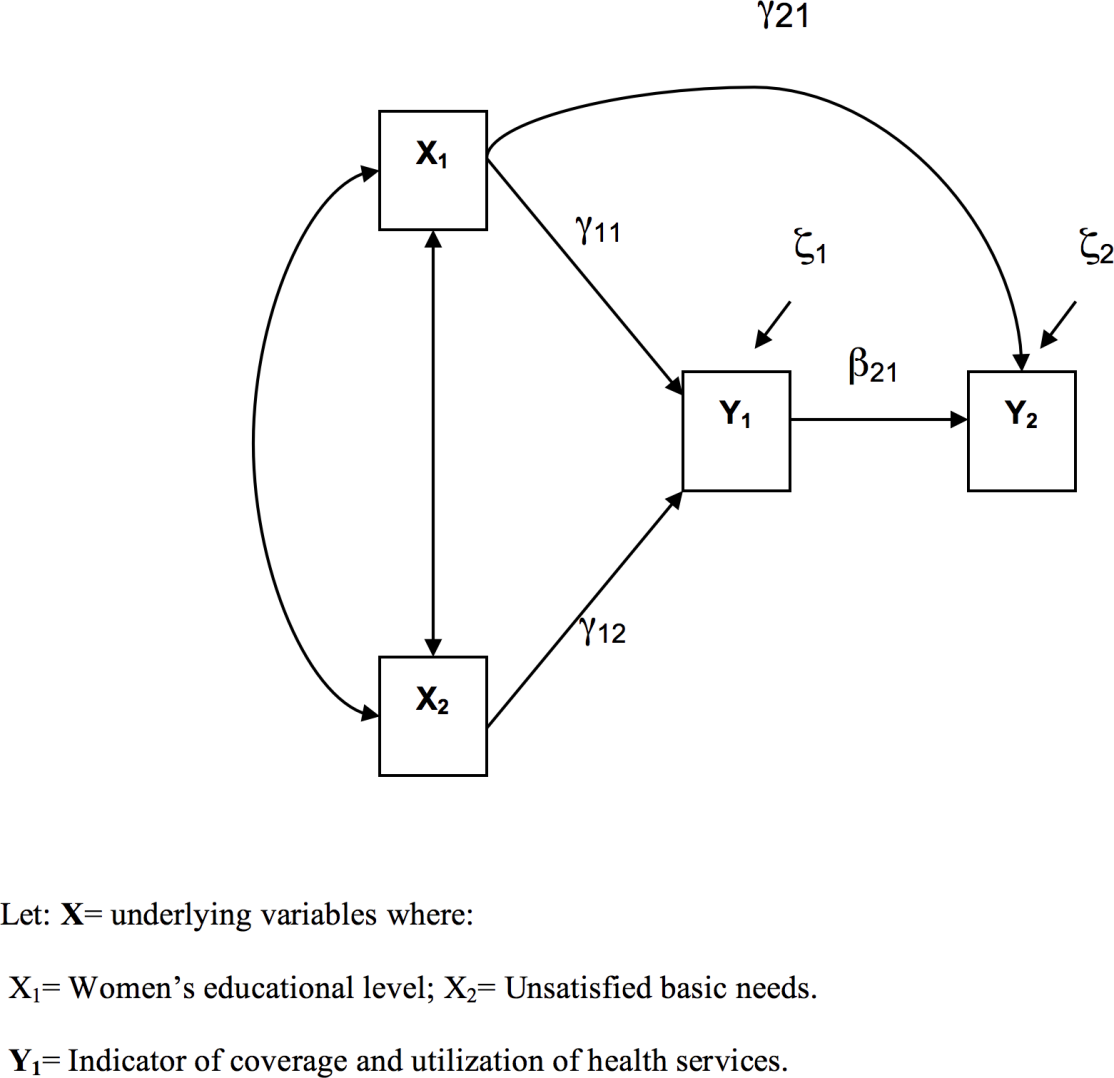
Model of the relationship among social determinants, health systems and health outcomes.

Fig 1 presents the initial model of the relationships among social determinants, where the healthcare system performance, which in this case refers to the indicator of coverage and utilization of maternal health services, is the mediating variable and the maternal mortality ratio is the final response variable. In this model, the variable used to construct the indicator of coverage and utilization of services was endogenous, and was mainly determined by the characteristics of the 33 states [35]. The path diagram shows that the underlying variables (social determinants) are exogenous. The proposed model is intended to reflect, in the most pragmatic way possible, the proposed conceptual model as well as the existing evidence of the problem under study [36].

The equation associated with the analytical model is:

$$Y_{1}=\gamma_{11}X_{1}+\gamma_{12}X_{2}+\varepsilon_{1}$$

$$Y_{2}=\gamma_{21}X_{1}+\beta_{21}Y_{1}+\varepsilon_{2}$$

The variables included in the model are described below.

#### Exogenous variables

This group includes the underlying variables that are present in the social structure and are potentially related to the performance of maternal health systems. The study variables were a) percentage of mothers who finished secondary school and b) the unsatisfied basic needs index.

#### Endogenous variables

The indicator of the coverage and utilization of maternal healthcare services was constructed using variables that evaluate the coverage and use of healthcare services, from prenatal check-ups to delivery and postpartum check-ups, as described earlier. The final response variable was the MMR.

All the data related to the variables were standardized according to the populations in the states. Associations with an alpha of 0.05 were considered statistically significant. The chi-square goodness-of-fit test was performed and the multiple correlation coefficient was estimated to validate the final model. All the analyses were performed using the Stata 13 statistical program (Stata Corporation, College Station, TX, USA).

## Results

### Factor analysis

**Fig 2:**
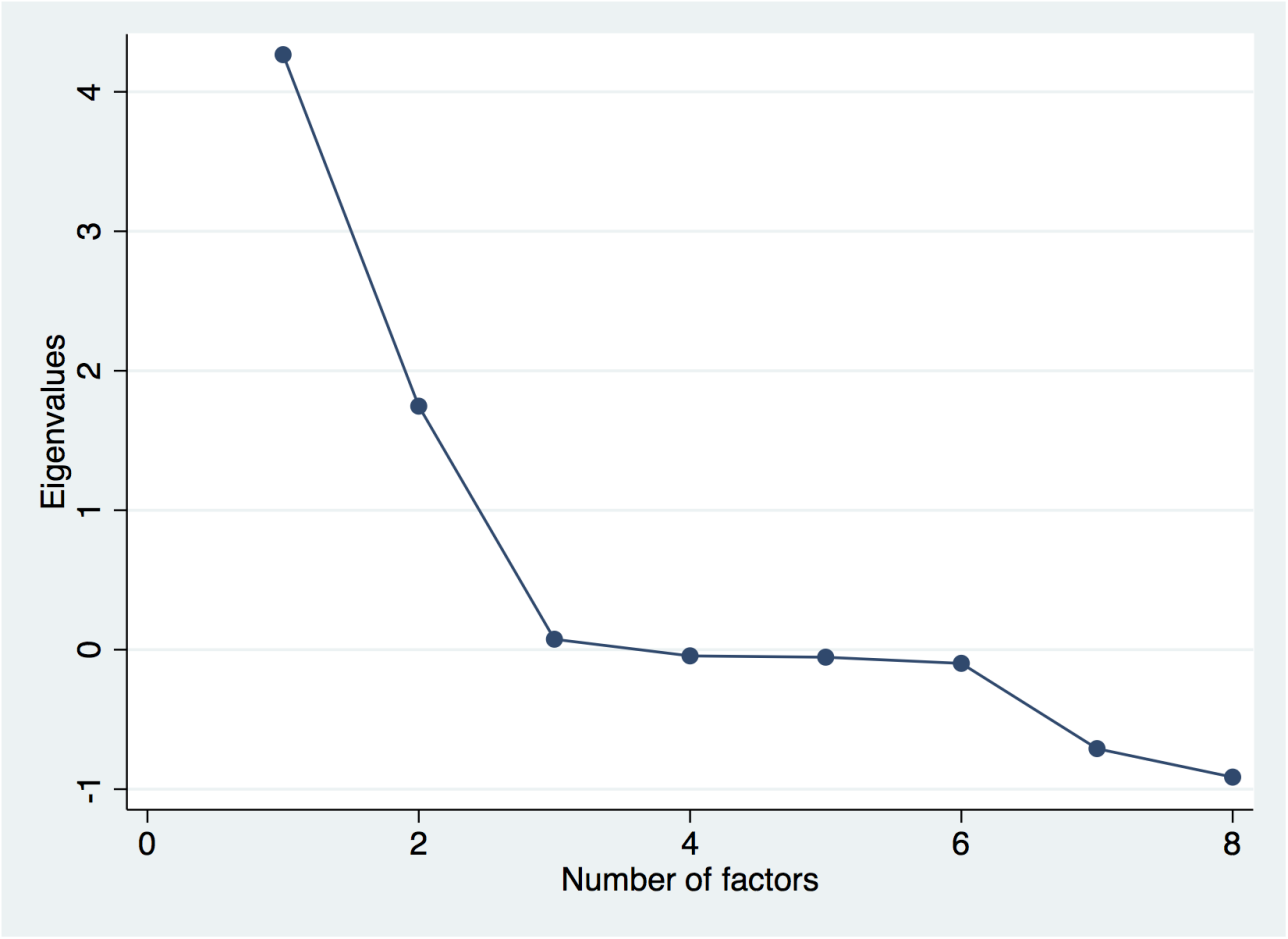
Scree plot of eigenvalues values after the principal components analysis.

The factor analysis presented in Table 1 indicates the existence of only one principal factor, which will be called “coverage and utilization of maternal healthcare services” (eigenvalue 4.35) (Fig 2). This is composed of 9 variables. Variables Q1 and Q4, which are related to institutionalized delivery care, are highly correlated with the principal factor (0.96 and 0.97, respectively). Variables Q3 and Q7, which characterize the lack of prenatal and postpartum health services, are inversely correlated with the principal factor (-0.86 and -0.96 respectively). Variable Q9, related to the establishment of a hospital infrastructure with care levels II and III, is highly correlated with the principal factor (with a correlation coefficient of 0.81). Finally, variables Q5 and Q6, reflecting financial coverage for prenatal and delivery care, are inversely correlated with the principal factor (Table 1).

**Table 1:** Factor loadings for the nine variables included in the indicator of coverage and utilization of maternal health services.

	Variable	F1	F2
Q1	% of births in a health institution	0.963	0.2393
Q2	% of reproductive-age women who currently use some type of modern birth control	0.7432	-0.1087
Q3	% of women who delivered within 5 years of the survey and did not receive prenatal care	-0.86	-0.1568
Q4	% of deliveries by a healthcare professional	0.9711	0.2136
Q5	% of pregnancies whose cost of prenatal care was not covered due to not being enrolled	-0.2385	0.922
Q6	% of pregnancies whose cost of the delivery was not covered due to not being enrolled	-0.1566	0.9464
Q7	% of pregnancies whose cost of postnatal care was not covered due to not being enrolled	-0.9633	-0.2047
Q8	% of deliveries by a midwife or family member	-0.4224	0.76
Q9	Second or third level hospital with obstetric services	0.8123	-0.21037

### Classification of states by quartiles of the indicator of coverage and utilization of maternal healthcare services

**Fig 3:**
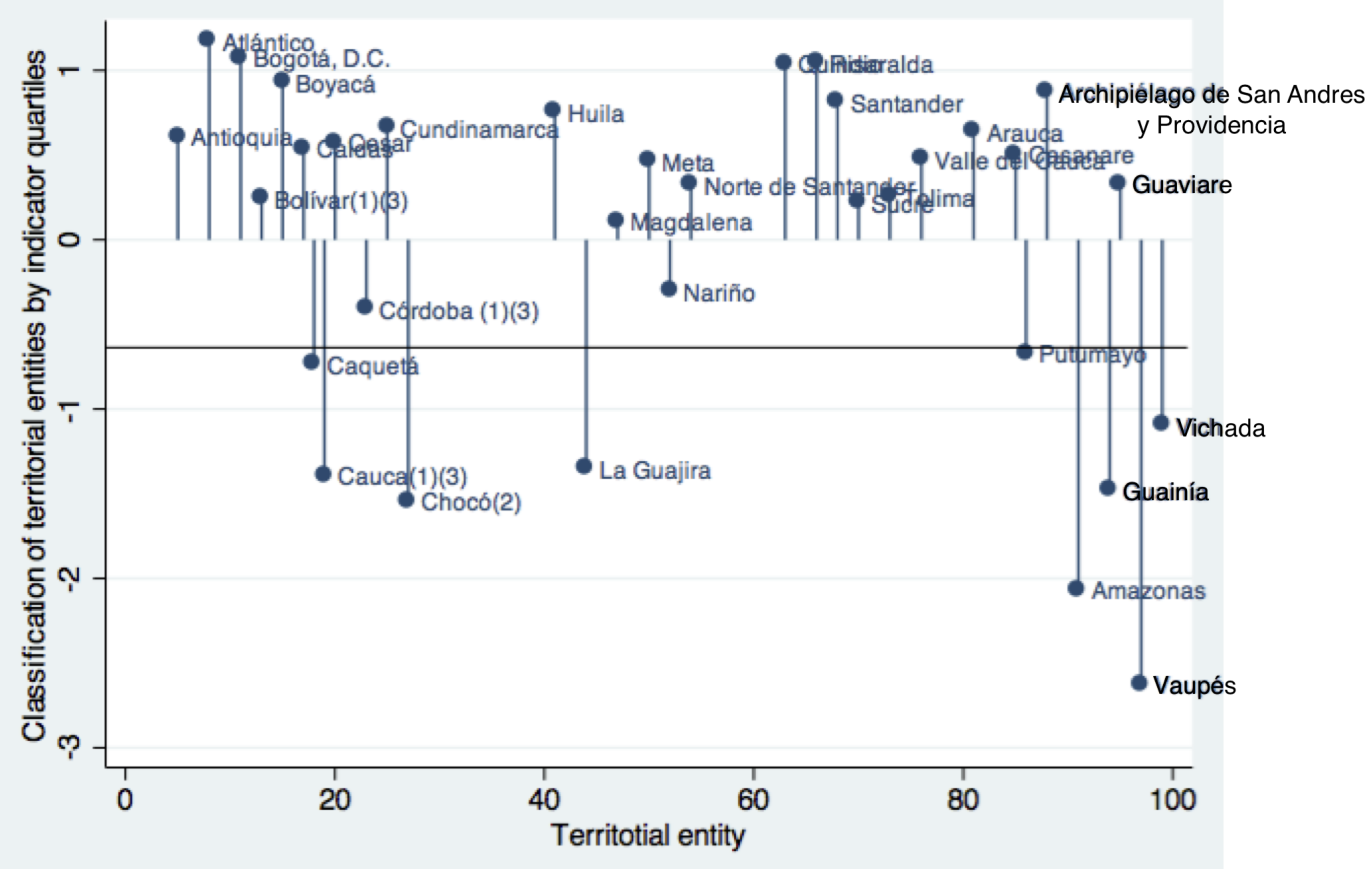
Classification of the territorial entities by quartile percentage of the indicator of coverage and utilization of maternal health services.

This indicator classified the states according to performance quartiles, with quartile 4 (Q4) as the best performance and quartile 1 (Q1) as the poorest. The states with the best performance were Atlantico, Bogota, Boyaca, Cundinamarca, Huila, Risaralda, Santander and San Andres. Those belonging to the Q1 category were Caqueta, Cauca, Choco, La Guajira, Vichada, Guainia, Amazonas and Vaupes. Fig 3 shows the classification of the states by quartiles of the indicator for the coverage and utilization of maternal healthcare services.

### Analysis of the relationships among social determinants, health system performance in maternal care and health outcomes

**Fig 4:**
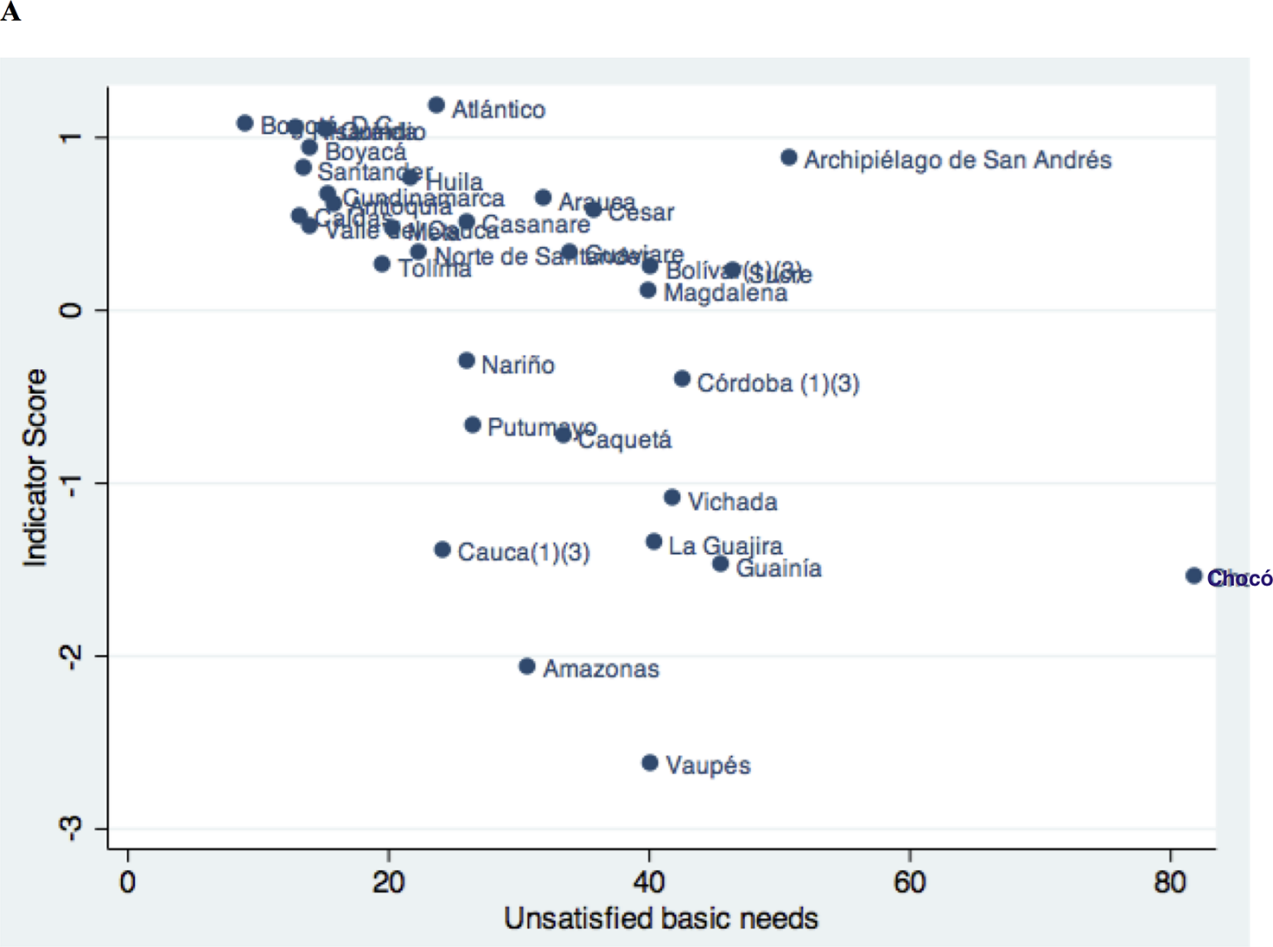
Behavior of the indicator of coverage and utilization of maternal health services in relation to social determinants.

A descriptive analysis was performed of the relationships between the social determinants and the indicator. An inverse relationship was identified between the unsatisfied basic needs index and the behavior of the indicator (rho = 0.16, *p* = 0.032), showing that the quality of maternal health is poorer when unsatisfied basic needs are greater. Meanwhile, a direct relationship was found with women’s education (rho = 0.71, *p* = 0.046), where populations with higher education levels were associated with higher quality scores (Fig 4).

**Fig 5:**
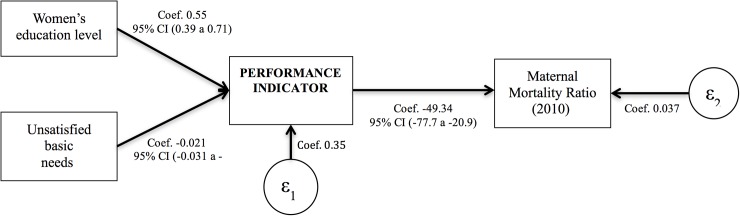
Path analysis of the maternal mortality ratio at the territorial entities level.

The path analysis presented a statistically significant association between the social determinants (unsatisfied basic needs index and the proportion of mothers who finished secondary school) and the behavior of the index generated for the coverage and utilization of maternal healthcare services (unsatisfied basic needs index *β* = -0.021; 95% CI: -0.031 to -0.01 and proportion of mothers who finished secondary school *β* = 0.554; 95% CI: 0.39 to 0.72). An inverse relationship was determined between the indicator generated and the behavior of the maternal mortality ratio, with a coefficient of *β* = -49.34 (95% CI: -77.7 to -20.9) (Table 2 and Fig 5).

**Table 2:** Path analysis: State-level maternal mortality ratio and indicator of healthcare system performance as a mediating variable. Colombia, 2010.

Structural	Coefficient	Standard Error	CI 95%		p
Unsatisfied basic needs (Department level)	-0.021	0.0051	-0.031	-0.01	<0.01
% education of women	0.554	0.083	0.39	0.72	<0.01
Constant	-4.14	0.815	-5.73	-2.54	<0.01
Maternal Mortality Ratio (2011)					
Performance indicator	-49.34	14.5	-77.77	-20.9	0.001
Constant	109.8	10.82	88.59	131.01	<0.01
Variance					
e. factor	0.347	0.116	0.18	0.67	
e. Maternal mortality ratio (2011)	3747.84	1467.64	1739.61	8074.38	

The path analysis model indicates that the healthcare system could function as a mediating variable between social determinants and a reduction in the maternal mortality ratio. The result from the chi-square goodness-of-fit test was 62.4 (*p* = 0.21) and the multiple correlation coefficient was 0.86, both of which validate the proposed model (Table 3).

**Table 3:** Tests to validate the path model.

	Model
No. of variables	4
Parameters estimated*	5
Goodness-of-fit *X*^*2*^	62.4 (*p* = 0.21)
Multiple correlation coefficients	0.86

*The number in parenthesis corresponds to the coefficients or correlations among variables.

## Discussion

The present study demonstrates the existence of a gap in the health system’s performance in the areas of coverage and utilization of maternal and reproductive health services in Colombia. The states most affected are Guainia, Choco, Vaupes, Amazonas and La Guajira, which according to other authors have the poorest social conditions and the highest maternal mortality rates [12]. The study herein provides two important contributions. First, it creates an indicator that evaluates the performance of healthcare systems in the areas of coverage and utilization of maternal health services in Colombia. This could be used in similar regions, both at the state level and the local level such as municipalities. Second, this work provides evidence of an association between the social determinants, poverty and women’s educational level, and the performance of the maternal and reproductive health system.

The indicator constructed by this study evaluated the coverage and utilization of healthcare services based on 9 variables. Nevertheless, it did not include an evaluation of the quality of the healthcare services because of a lack of state-level indicators to measure this aspect of the health system. In spite of this limitation, since the indicator is able to explain the behavior of the maternal mortality ratio, it can identify state-level differences in the functioning of coverage and utilization. A conditional relationship among the functioning of coverage, utilization and quality could be assumed, the latter being a product of the first two, as reported by Andersen [36, 37]. The indicator also could be used to determine inequities at different regional levels, both within as well as among countries, making it possible to identify the areas having the greatest need for reforming the healthcare system, according to their particular contexts.

Other indicators have been developed to evaluate the utilization and quality of maternal health services. For example, in 2011, Rahman M, *et al*. [38] published the development of an indicator that assessed inequities (on the national level) in the provision of healthcare services between the rich and poor in the Republic of Vanuatu. The authors concluded that access to health services provided by a healthcare professional during delivery was the factor that could contribute to the greatest inequity. In 2005, Laditka SB, *et al*. [39] developed an indicator which evaluated access to primary maternal health services and prenatal care at the national level. They concluded that effective access to more than one prenatal check-up reduces the risk of complications during delivery. In addition, Nagraj S, *et al*. 2013 [40] constructed an indicator for the United Kingdom which evaluated the utilization and quality of health services provided by primary health care programs. Although this indicator evaluated attitudes and perceptions related to health behaviors in the home, it identified a good patient-physician relationship and the perception of trust as factors associated with better access to the healthcare system and satisfactory health outcomes.

Several studies have reported that the main causes of inequitable utilization of maternal healthcare services among populations are poverty, low education level and difficulty gaining access to healthcare services due to geographic factors or differences in the quality of services [12,41,42]. In the specific case of Colombia, the National Health Observatory reported that healthcare services appear to play a determinant role in maternal mortality, especially for social groups such as women from rural areas, ethnic minorities, the unemployed and those in poverty [14]. In addition, four studies (including one on the national level) showed the poor to be the most disadvantaged group with regard to maternal health services in Colombia [43–46], particularly those with unsatisfied basic needs such as housing and access to healthcare services [45,46]. And a study at the municipal level demonstrated a statistically significant relationship between the MMR and the multidimensional poverty index (risk ratio of 3.52; 95% CI: 1.09–11.38) [12]. The above findings are consistent with the first component in the path analysis presented herein, which indicates that the socioeconomic level of a region is a determinant of maternal mortality, except that the present work suggests that this relationship may be mediated by the functioning of the healthcare system.

The quality of healthcare services and health outcomes in Colombia are heterogeneous [12,14–16]. In addition to poverty, women’s educational level is a contextual variable which is also an important structural determinant of the inequities in the quality of maternal healthcare in the country. At the local level, this may be due to cultural barriers to access, as reported in 2012 by Singh PK and colleagues [47] who found that low educational levels on the part of the mother and partner are associated with a higher occurrence of teenage pregnancy. Meanwhile, from a broader macro-social perspective, a higher educational level on the part of women may be related to a demand for better healthcare services [48–50] in a particular region, since a higher educational level can have the effect of empowering women, and this has been correlated with several positive health outcomes, possibly including a decrease in maternal mortality [48].

The evidence provided by this work demonstrates that health inequities can be identified even within a country operating under a single healthcare system. These inequities are explained by social determinants and directly affect how a single system may perform within a region. These relationships have been explored by earlier studies that demonstrated that social determinants of health (such as less ethnic and linguistic fragmentation, a high degree of democracy and acceptable educational coverage for women) are related to better maternal health outcomes at the national level [51]. Thus, it is possible to identify the ways in which national and sub-national systems need to be adapted to particular social, cultural, and health contexts in order to create effective public policies and interventions that address social determinants of health.

While several theories exist about why social inequities are determinants of the health status of populations, the study herein explored a possible relationship between social determinants and the performance of healthcare systems, as well as how this relationship can result in an inadequate response to the health needs of populations [52]. This opens the door to exploring possible theories that may shed light on the functioning of a healthcare system and its actors, such as social capital theory and its relationship to the effective utilization of healthcare services. For example, some authors have found positive effects in environments that favor social growth and that support communication networks among the different actors in social and healthcare systems [53,54]. In England, high social capital was found to contribute to the health of its citizens and to a reduction in health inequities, where social capital was measured by the opportunity for civic participation in decision-making processes and political efficiency. In addition, an increase in women’s cognitive social capital has been shown to improve children’s health as a result of better adherence to healthy lifestyle habits and timely use of healthcare services [55–58].

The strengths of this study include its methodological design, which enabled establishing relationships among social determinants, the aggregated variables used to evaluate the health system and the maternal mortality ratio at the state level. Meanwhile, a limitation of the study may be the possibility of bias due to confounding variables that were not measured, since other macroeconomic and social variables and the performance of the system were not included. For example, social capital, democratization and corruption relate to other processes and observation levels that could not be measured by this study. Therefore, the results of the present study should be carefully interpreted. The model presented should also be replicated in diverse contexts and at different regional levels.

## Conclusions

Improving access to modern family planning methods and institutionalizing delivery and healthcare provided by trained professionals are strategies that have had a positive impact worldwide [47]. Nevertheless, gaps in coverage and in the utilization of maternal health services exist in Colombia. The states of Choco, Guainia, Amazonas and La Guajira present the greatest deficiencies, with a negative impact on maternal health, as compared to Bogota, Atlantico and Santander which have better processes to provide healthcare services and thus have favorable health outcomes. The indicator constructed to evaluate the coverage and utilization of maternal health services is suitable for assessing this aspect of the healthcare system. It was also found to behave adequately at the state level and to be sensitive to social changes. Therefore, it can serve as an appropriate instrument to measure the functioning of the Colombian healthcare system in the area of maternal health. In addition, a possible relationship was established between social determinants and how well the health system provided healthcare services, where the performance of the system functions as a mediating factor between social determinants and health outcomes. Future studies to explore and evaluate this relationship will be worthwhile.

## Supporting information

S1 Appendix Territorial entities database. (XLSX)
