# Correction: Generation and evaluation of an indicator of the health system's performance in maternal and reproductive health in Colombia: An ecological study

**DOI:** 10.1371/journal.pone.0187534

**Published:** 2017-10-30

**Authors:** The PLOS ONE Staff

## Notice of Republication

This article was republished on September 5, 2017, to correct an error in the first author’s name that was introduced during the typesetting process. The publisher apologizes for the error.
